# Capsular Polysaccharide Cross-Regulation Modulates Bacteroides thetaiotaomicron Biofilm Formation

**DOI:** 10.1128/mBio.00729-20

**Published:** 2020-06-23

**Authors:** Nathalie Béchon, Jovana Mihajlovic, Sol Vendrell-Fernández, Florian Chain, Philippe Langella, Christophe Beloin, Jean-Marc Ghigo

**Affiliations:** a Institut Pasteur, Genetics of Biofilms Laboratory, Paris, France; b Université de Paris, Ecole Doctorale Bio Sorbonne Paris Cite (BioSPC), Cellule Pasteur, Paris, France; c Commensals and Probiotics-Host Interactions Laboratory, Université Paris-Saclay, INRAE, AgroParisTech, Micalis Institute, Jouy-en-Josas, France; University of Minnesota Medical School

**Keywords:** capsule, *Bacteroides thetaiotaomicron*, biofilm

## Abstract

Bacteroides thetaiotaomicron is one of the most abundant gut symbiont species, whose contribution to host health through its ability to degrade dietary polysaccharides and mature the immune system is under intense scrutiny. In contrast, adhesion and biofilm formation, which are potentially involved in gut colonization and microbiota structure and stability, have hardly been investigated in this intestinal bacterium. To uncover B. thetaiotaomicron biofilm-related functions, we performed a transposon mutagenesis in the poorly biofilm-forming reference strain VPI-5482 and showed that capsule 4, one of the eight B. thetaiotaomicron capsules, hinders biofilm formation. We then showed that the production of capsules 1, 2, 3, 5, and 6 also inhibits biofilm formation and that decreased capsulation of the population correlated with increased biofilm formation, suggesting that capsules could be masking adhesive surface structures. In contrast, we showed that capsule 8 displayed intrinsic adhesive properties. Finally, we demonstrated that *BT2934*, the *wzx* homolog of the B. thetaiotaomicron glycosylation locus, competes with capsule production and impacts its adhesion capacity. This study therefore establishes B. thetaiotaomicron capsule regulation as a major determinant of B. thetaiotaomicron biofilm formation, providing new insights into how modulation of different B. thetaiotaomicron surface structures affects *in vitro* biofilm formation.

## Abstract (precis)

The human gut harbors a complex bacterial community that plays important roles in host health and disease, including nutrient acquisition, maturation of the immune system, and resistance to infections. The capacity to adhere to surfaces and form communities called biofilms is believed to be important for niche colonization and maintenance of gut bacteria. However, little is known about the adhesion capacity of most gut bacteria. In this study, we investigated biofilm formation in Bacteroides thetaiotaomicron, one of the most abundant bacteria of the normal mammalian intestine. We identified that B. thetaiotaomicron capsules, a group of eight surface-exposed polysaccharidic layers mediating important interactions with the gut environment, are also major determinants of biofilm formation that mask or unmask adhesion factors. Studying how B. thetaiotaomicron regulates its adhesion properties will allow us to better understand the physiology and specific properties of this important gut symbiont within anaerobic biofilms.

## INTRODUCTION

Bacteroides thetaiotaomicron is an abundant bacterial symbiont of the normal mammalian intestine that contributes to shaping the nutrient environment of the gut microbiome through degradation of complex polysaccharides and production of short-chain fatty acids (1–5). B. thetaiotaomicron was also shown to stimulate the development of gut immunity (6), attenuate intestinal inflammation (7), and strengthen the intestinal protective barrier (8, 9). Consistently, a decrease in the abundance of B. thetaiotaomicron and other *Bacteroides* species has been correlated with gut inflammation and disease emergence, underlining the importance of the gut microbiota for host intestinal physiology and health (10). In contrast, microbial functions involved in the establishment and maintenance of a healthy gut microbiota are still not well understood. It is speculated that the ability of symbiont bacteria to form biofilms could contribute to microbiota stability (11, 12). However, although bacterial biofilm formation has been studied in various facultative symbiotic and pathogenic anaerobes, information on this widespread lifestyle is still scarce in B. thetaiotaomicron (13–15). Whereas comparative gene expression profiling between biofilm and planktonically grown B. thetaiotaomicron showed biofilm-associated upregulation of polysaccharide utilization systems and capsule 8, one of the eight B. thetaiotaomicron capsule synthesis loci (15, 16), there is still no direct proof of the contribution of these surface structures to adhesion and biofilm formation. We recently showed that although biofilm capacity is widespread among B. thetaiotaomicron isolates, the widely used reference strain VPI-5482 is a poor biofilm former. Nevertheless, use of transposon mutagenesis followed by a positive selection procedure revealed mutants with significantly improved biofilm capacity due to alteration of the structure of a putative type V pilus (13). Here, we identified two mechanisms of capsule regulation in B. thetaiotaomicron, involving UpxZ-mediated capsule transcription inhibition and competition between protein glycosylation and capsule production. We showed that capsule masking or unmasking of adhesive structures is a major determinant of B. thetaiotaomicron biofilm formation. This study therefore provides new insights into the roles of capsular polysaccharides in B. thetaiotaomicron and their impact on the physiology and biofilm formation of a prominent gut symbiont.

## RESULTS

### Transposon insertion in the capsule 4 biosynthesis operon promotes B. thetaiotaomicron biofilm formation.

**FIG 1:**
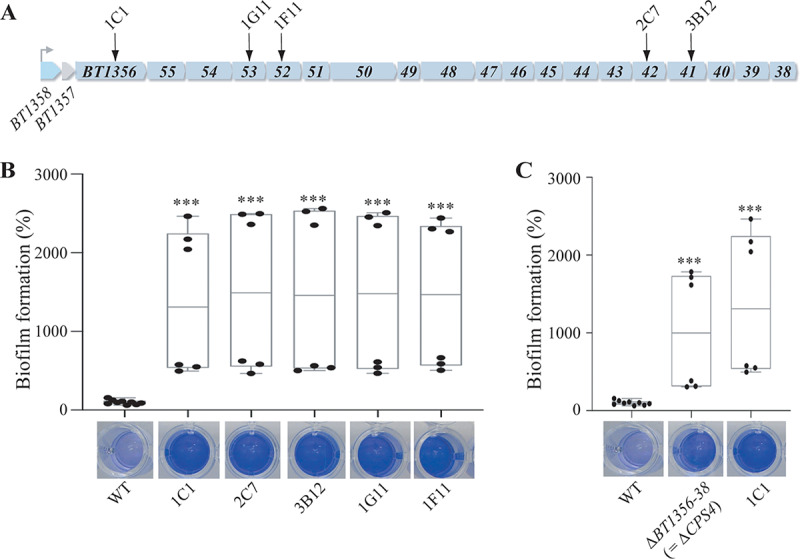
Capsule 4 inhibits biofilm formation in B. thetaiotaomicron VPI-5482. (A) Organization of B. thetaiotaomicron capsular operon 4 (*CPS4*). The first two genes (*BT1358* and *BT1357*) code regulators of capsular biosynthesis. *BT1356-1338* code the enzymes involved in Cps4 capsular polysaccharide biosynthesis. The arrows indicate 5 individual transposon insertions within the *CPS4* operon. (B) The 96-well plate biofilm assay after 48 h of growth in BHIS. The Mean of the WT is adjusted to 100%. In the min-max boxplot of 6 biological replicates for each strain, each replicate is the mean of two technical replicates. ***, *P* < 0.0005; Mann-Whitney test, comparing the indicated mutant to the WT. (C) The 96-well plate biofilm assay after 48 h of growth in BHIS. The mean of the WT is adjusted to 100%. In the min-max boxplot of 6 to 9 biological replicates for each strain, each replicate is the mean of two technical replicates. ***, *P* < 0.0005; Mann-Whitney test, comparing the indicated mutant to the WT. The images shown under each boxplot correspond to representative CV-stained microtiter wells after resuspension of the biofilm.

Among the previously identified transposon mutants displaying increased *in vitro* biofilm formation capacity compared to the wild-type B. thetaiotaomicron VPI-5482 (WT) (13), five of them corresponded to insertions within capsule 4 (*CPS4*) synthesis operon *BT1358-1338*, encoding one of the eight capsular polysaccharides of B. thetaiotaomicron (Fig. 1A and B; see Table S1 in the supplemental material) (16, 17). To confirm the increased biofilm phenotype of the transposon mutants, we deleted all 19 *CPS4* structural genes located downstream of the regulators *BT1358-1357*. Crystal violet staining of *in vitro* biofilm formed in 96-well microtiter plates showed that the resulting *ΔBT1356-1338* mutant (here named *ΔCPS4*) displayed a significant increase in biofilm formation compared to the wild-type B. thetaiotaomicron VPI-5482 (Fig. 1C).

10.1128/mBio.00729-20.6 TABLE S1 Transposon insertion targets. Download Table S1, PDF file, 0.1 MB. Copyright © 2020 Bechon et al. 2020 Bechon et al. This content is distributed under the terms of the Creative Commons Attribution 4.0 International license.

### B. thetaiotaomicron biofilm formation is modulated by capsule cross-regulation.

**FIG 2:**
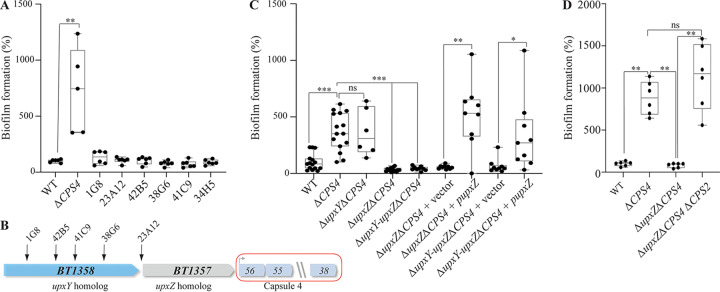
Capsule cross-regulation modulates biofilm formation in B. thetaiotaomicron. (A) The 96-well plate crystal violet biofilm assay after 48 h of growth in BHIS. (B) Organization of B. thetaiotaomicron capsular operon 4 (CPS4) with identified transposon insertion points in the first two genes of the operon (*BT1358* and *BT1357*), coding regulators of capsular biosynthesis. (C) The 96-well plate crystal violet biofilm assay after 48 h of growth in BHIS. The Mean of the WT is adjusted to 100%. (D) The 96-well plate crystal violet biofilm assay after 48 h of growth in BHIS. In panels A, C, and D, the mean of the WT is adjusted to 100%. In the min-max boxplot of 6 to 9 biological replicates for each strain, each replicate is the mean of two technical replicates. **, *P* < 0.005; Mann-Whitney test. *upxY*, *upxY^BT1358^*; *upxZ*, *upxZ^BT1357^*.

To uncover the mechanism of increased biofilm formation in a *ΔCPS4* strain, we performed a random transposon mutagenesis in Δ*CPS4* and identified 6 mutants out of 4,650 with reduced biofilm formation capacity compared to the parental *ΔCPS4* (Fig. 2A). Five of these mutants corresponded to transposons inserted in the *BT1358*-*1357* region just upstream of the CPS4 operon (Fig. 1A and Fig. 2B; Table S1). *BT1358* codes for an UpxY-like homolog and *BT1357* codes for a UpxZ-like homolog, two regulatory genes located at the beginning of most capsule synthesis operons in B. thetaiotaomicron and Bacteroides fragilis (18, 19). UpxY-like proteins positively regulate their cognate capsular operon by preventing premature transcription termination in the untranslated region, thus facilitating the otherwise abortive transcription of the downstream capsular genes (18). In contrast, UpxZ-like proteins are repressors of transcription of nonadjacent capsular systems (19). We first showed that deletion of *upxY^BT1358^* in B. thetaiotaomicron *ΔCPS4* did not impact biofilm formation, which is consistent with its role as a positive regulator of the expression of capsule 4 genes, all missing in the *ΔCPS4* mutant (Fig. 2B and C). We then hypothesized that transposon insertion in *upxY^BT1358^* (located upstream of *upxZ ^BT1357^*) could have a polar effect on the expression of the repressor *upxZ^BT1357^*, leading to the derepression of one or more of the 7 other B. thetaiotaomicron capsular polysaccharides. Indeed, in-frame deletion of *upxZ^BT1357^* or *upxY^BT1358^-upxZ^BT1357^* in a Δ*CPS4* background did not affect growth but led to loss of biofilm capacity (Fig. 2C and Fig. S1A). This phenotype could be complemented in *trans* by introducing *upxZ^BT1357^* expressed from a constitutive promoter in the 5′ untranslated region of the tRNA-Ser chromosomal locus, in either a Δ*upxZ^BT1357^* Δ*CPS4* or Δ*upxY^BT1358^-upxZ^BT1357^* Δ*CPS4* B. thetaiotaomicron background (Fig. 2C). To identify which capsules were repressed by *upxZ^BT1357^*, we used reverse transcription-quantitative PCR (qRT-PCR) to monitor the expression of each capsular operon, and we observed an increased transcription of capsule 2 (CPS2) in B. thetaiotaomicron Δ*upxZ^BT1357^* Δ*CPS4* compared to the B. thetaiotaomicron Δ*CPS4* single mutant (Fig. S2). Consistently, deletion of the CPS2 operon in the B. thetaiotaomicron Δ*upxZ^BT1357^* Δ*CPS4* background restored biofilm formation capacity (Fig. 2D) Thus, expression of either CPS4, or CPS2 in the absence of CPS4, hinders biofilm formation.

10.1128/mBio.00729-20.1 FIG S1 The mutants considered in this study were not affected for growth. Planktonic growth of the indicated mutants for 24 h in BHIS broth in 96-well plates. The OD_600_ was measured every 30 min. Each dot is the median of 6 biological replicates. Error bars represent the 95% confidence interval. (A) Impact of *BT2934-2938* on growth. (B) *ΔCPS1-8* transposon mutants. Download FIG S1, TIF file, 2.5 MB. Copyright © 2020 Bechon et al. 2020 Bechon et al. This content is distributed under the terms of the Creative Commons Attribution 4.0 International license.

10.1128/mBio.00729-20.2 FIG S2 *BT1357* is a repressor of capsule 2 transcription. qRT-PCR of 3 biological replicates of overnight planktonic cultures of WT, *ΔCPS4*, *ΔCPS4ΔupxZ^BT1357^* following one gene of each capsular operon and 16sRNA and *rpoB* housekeeping genes. (A) Expression levels of each capsule were controlled within each strain using the expression of 16s rRNA and *rpoB* housekeeping genes, and they were normalized between strains using the expression of the WT as a reference. The *y* axis shows the fold-change of expression (2^-ΔΔCt^) for each of the 8 capsules of B. thetaiotaomicron. (B) Expression levels of each capsule normalized by 16s RNA and *rpoB* genes in the WT. The *y* axis shows the fold-change of expression within one strain (2^-ΔCt^) for each of the 8 capsules of B. thetaiotaomicron. Download FIG S2, TIF file, 2.5 MB. Copyright © 2020 Bechon et al. 2020 Bechon et al. This content is distributed under the terms of the Creative Commons Attribution 4.0 International license.

### Expression of capsule 8 and lack of any capsules both induce biofilm formation.

**FIG 3:**
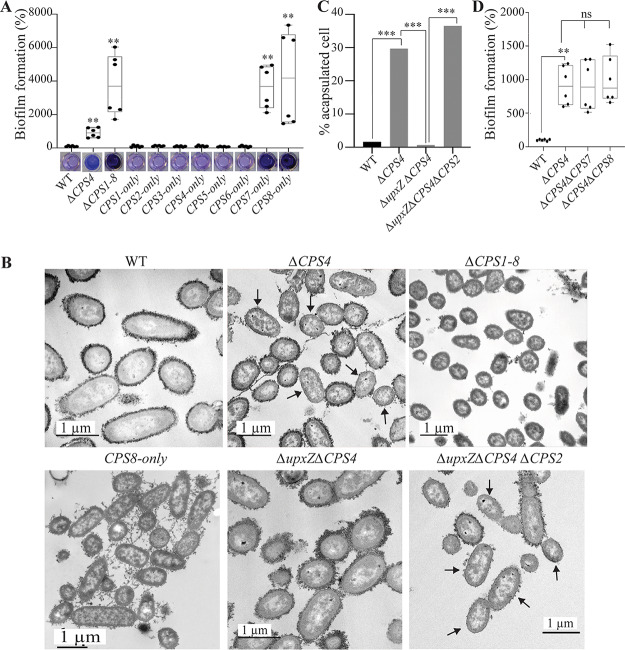
Capsule expression in B. thetaiotaomicron is heterogenous and has consequences for biofilm formation. (A and D) The 96-well plate biofilm assay after 48 h of growth in BHIS. The mean of the WT is adjusted to 100%. In the min-max boxplot of 6 biological replicates for each strain, each replicate is the mean of two technical replicates. **, *P* < 0.005; Mann-Whitney test, comparing the indicated mutant to the WT. The pictures shown under the boxplot in panel A correspond to representative CV-stained microtiter wells after resuspension of the biofilm. (B) Transmission electron microscopy (TEM) images of overnight cultures fixed with ferritin. Arrows indicate some examples of acapsulated cells. (C) Percentage of acapsulated cells of indicated strains counted on TEM pictures. For each strain, at least 100 cells were counted. ***, *P* < 0.0005; prop.test (R). *upxY*, *upxY^BT1358^*; *upxZ*, *upxZ^BT1357^*.

To assess the contribution of all capsules, besides inhibition by CPS4 or CPS2, to B. thetaiotaomicron biofilm formation, we used a recently described set of strains expressing only one of the eight B. thetaiotaomicron capsular types (20). We observed that derivative strains expressing only capsule 1, 2, 3, 4, 5, or 6 formed as little biofilm as wild-type (WT) B. thetaiotaomicron VPI-5482. Interestingly, strains only expressing CPS7 or CPS8 formed over 35 times more biofilm than the WT strain (Fig. 3A). However, all CPS7-only bacteria seemed to be acapsulated, which is consistent with previous observations suggesting that capsule 7 may not be expressed in tested laboratory conditions (Fig. S3A and B) (20). Indeed, similar to a CPS7-only strain, a strain deleted for all 8 capsule operons (*ΔCPS1-8*) formed 40 times more biofilm than the WT (Fig. 3A) and showed a strong aggregation phenotype in overnight cultures (Fig. S3B). In contrast, India ink staining confirmed the presence of a capsule in biofilm-forming (but not aggregating) CPS8-only bacteria, suggesting that capsule 8 could have intrinsic adhesive properties (Fig. S3A and B). These results showed that, except for CPS8 and potentially CPS7, the expression of all capsules hinders B. thetaiotaomicron biofilm formation and that acapsulated cells display cell-to-cell aggregation capacity likely driving the observed increased biofilm phenotype.

10.1128/mBio.00729-20.3 FIG S3 Acapsulated B. thetaiotaomicron aggregate in BHIS. (A) India ink-stained cultures observed under the phase contrast microscope, ×1000. (B and C) Aggregation in overnight cultures of B. thetaiotaomicron in BHIS broth. (D) Observation of bacterial aggregates with a phase contrast microscope, ×400. Download FIG S3, TIF file, 2.4 MB. Copyright © 2020 Bechon et al. 2020 Bechon et al. This content is distributed under the terms of the Creative Commons Attribution 4.0 International license.

### Deletion of capsule 4 leads to a heterogeneously capsulated bacterial population.

To determine whether lack of capsule or expression of the biofilm-promoting capsule 8 was responsible for the observed increased biofilm formation in the *ΔCPS4* strain, we used transmission electron microscopy (TEM) and showed that whereas WT B. thetaiotaomicron bacteria were almost all capsulated (>98%), ca. 30% of *ΔCPS4* cells lacked a visible capsule (Fig. 3B and C). Considering that *ΔCPS1-8* formed 4 times more biofilm than Δ*CPS4*, this suggested a correlation between the increased frequency of noncapsulated cells in the population and the increased ability to form biofilms (Fig. 3A and C). To determine whether capsulated cells in the *ΔCPS4* population contributed to adhesion, we deleted, in the *ΔCPS4* background, either *CPS8*, the only biofilm-promoting capsule of B. thetaiotaomicron, or *CPS7*, for which we could not ascertain the biofilm formation potential using a single CPS-expressing strain. Both the *ΔCPS4 ΔCPS7* and *ΔCPS4 ΔCPS8* mutants had similar biofilm capacity compared to a *ΔCPS4* mutant, showing that neither capsule 7 nor 8 contribute to biofilm formation in the absence of capsule 4 (Fig. 3D). Moreover, TEM imaging showed that the non-biofilm-forming Δ*upxZ^BT1357^* Δ*CPS4* double mutant was entirely capsulated (due to induction of CPS2; Fig. S2), supporting a correlation between increased biofilm formation (Fig. 3A) and the presence of a subpopulation of acapsulated cells in the *ΔCPS4* strain (Fig. 3B and C). Consistently, deletion of *CPS2* in the Δ*upxZ^BT1357^* Δ*CPS4* background led to the emergence of 37% of acapsulated bacteria in a Δ*upxZ^BT1357^* Δ*CPS4ΔCPS2* population (Fig. 3B and C) and restored biofilm formation (Fig. 2D).

### Identification of BT2934 as a new B. thetaiotaomicron inhibitor of capsule expression.

**FIG 4:**
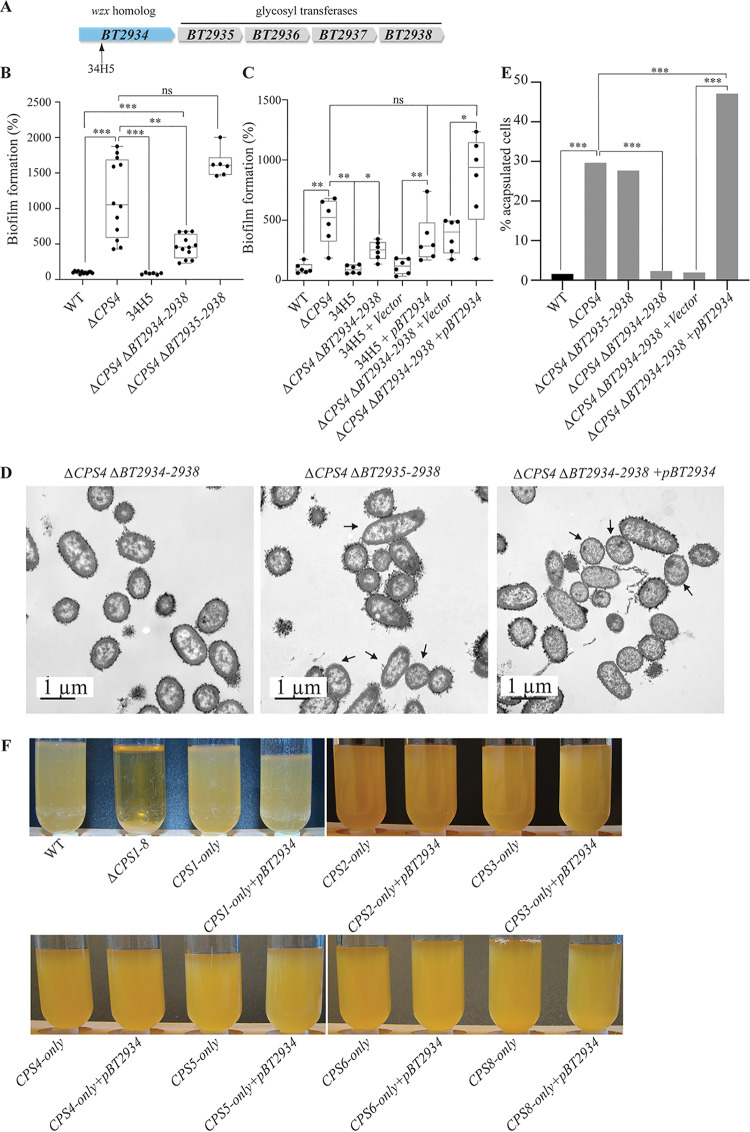
*BT2934* is a novel capsule inhibitor. (A) Organization of the B. thetaiotaomicron protein glycosylation *BT2934* locus with identified transposon insertion point. (B and C) The 96-well plate crystal violet biofilm assay after 48 h of growth in BHIS. The mean of the WT is adjusted to 100%. In the min-max boxplot of 6 to 12 biological replicates for each strain, each replicate is the mean of two technical replicates. *, *P* < 0.05; **, *P* < 0.005; ***, *P* < 0.0005; Mann-Whitney test. (D) TEM images of *ΔCPS4ΔBT2934-2938*, *ΔCPS4ΔBT2935-2938*, and *ΔCPS4ΔBT2934-2938+pBT2934* overnight cultures fixed with ferritin. The arrows indicate some acapsulated cells as examples. (E) Percentage of acapsulated cells in overnight cultures counted on TEM pictures. For each strain, at least 100 cells were counted. ***, *P* < 0.0005; prop.test (R). (F) Overnight cultures of indicated strains in BHIS. Only ΔCPS1-8 showed aggregation.

In addition to mutation in Δ*upxZ ^BT1357^* capsule repressor, we also identified a biofilm-deficient *ΔCPS4* transposon mutant (34H5) with an insertion in *BT2934* (Fig. 3A and 4A). The *BT2934-2947* region corresponds to a B. thetaiotaomicron protein glycosylation locus (21, 22), in which *BT2934* encodes a homolog of the transmembrane oligosaccharide flippase Wzx (Fig. 4A). We deleted *BT2934* and the 4 putative glycosyl transferase genes, *BT2935-2938*, located in the same operon and confirmed the role of *BT2934-2938* in protein glycosylation, as several bands disappeared from a protein glycosylation profile in the *ΔCPS4 ΔBT2934-2938* and 34H5 mutants compared to *ΔCPS4* (Fig. S4). The double mutant Δ*CPS4* Δ*BT2934-2938* had no growth defect and displayed a 2-fold decrease in biofilm formation compared to Δ*CPS4* (Fig. 4B; Fig. S1B). However, it still formed more biofilm than the original 34H5 transposon mutant in *BT2934*. To determine the origin of this discrepancy, we only deleted *BT2935-2938* glycosyl transferases genes and did not observe reduced biofilm capacity compared to the *ΔCPS4* strain. Although we did not succeed in deleting *BT2934* alone, introduction of p*BT2934*, constitutively expressing *BT2934*, in the 34H5 transposon mutant and *ΔCPS4 ΔBT2934-38* mutant restored biofilm formation but still showed an altered protein glycosylation profile (Fig. 4C; Fig. S4). These results suggested that the impact of *BT2934* on biofilm formation did not involve *BT2935-2938* and might not directly involve protein glycosylation. Finally, we showed that while *ΔCPS4* and *ΔCPS4 ΔBT2935-2938* bacteria displayed similar level of acapsulated cells (30% and 28%, respectively), *ΔCPS4 ΔBT2934-2938* cells showed full, wild-type levels of capsulation (Fig. 4D and E), reduced back down to over 50% of capsulated cells upon complementation by p*BT2934* (Fig. 4D and E). To identify whether *BT2934* directly inhibited capsule production, we overexpressed *BT2934* in each strain only expressing a single CPS type. We hypothesized that overexpression of BT2934 in each of these strains could inhibit capsule expression and lead to acapsulation of the whole population, thus leading to aggregation in overnight cultures. However, none of the resulting strains aggregated, suggesting that no capsules were directly inhibited by an overexpression of *BT2934.* Taken together, these results suggest that *BT2934* indirectly impacts capsule production in B. thetaiotaomicron, with consequences affecting its ability to form biofilm.

10.1128/mBio.00729-20.4 FIG S4 *BT2934-2938* is involved in protein glycosylation. ProQ Emerald 300 staining of glycosylated proteins on whole-cell extract of overnight cultures. Download FIG S4, TIF file, 2.6 MB. Copyright © 2020 Bechon et al. 2020 Bechon et al. This content is distributed under the terms of the Creative Commons Attribution 4.0 International license.

### Biofilm-forming *CPS4* and *BT2934* mutants are outcompeted by the wild-type strain *in vivo*.

**FIG 5:**
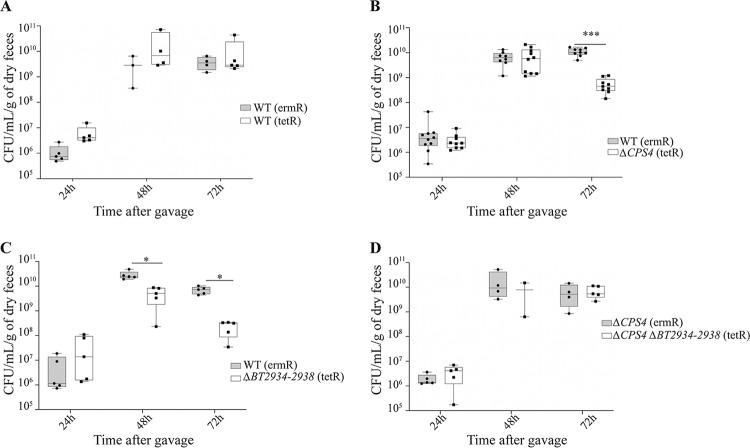
*BT2934* and *CPS4* contribute to *in vivo* colonization in axenic mice. Min-max boxplot of CFU/ml/dry weight of feces, numbered from feces from 5 to 10 axenic mice after cocolonization with indicated strains. *, *P* < 0.05; **, *P* < 0.005; ***, *P* < 0.0005; Mann-Whitney test. (A) WT (ermR) versus WT (tetR). (B) WT (ermR) versus *ΔCPS4* (tetR). (C) (ermR) versus *ΔBT2934-2938* (tetR). (D) *ΔCPS4* (ermR) versus *ΔCPS4ΔBT2934-2938* (tetR).

*CPS4* and *BT2934* have previously been shown to be important for *in vivo* colonization in the presence of a complex mix of B. thetaiotaomicron transposon mutants (23). To test whether unmasking B. thetaiotaomicron biofilm formation capacity could contribute to *in vivo* colonization, we used intragastric gavage to inoculate axenic mice with erythromycin-resistant WT-*erm*- and tetracycline-resistant *ΔCPS4-tet or ΔBT2934-38-tet* in a 1:1 mix ratio and measured the abundance of each strain in feces for 8 days using erythromycin and tetracycline resistance to discriminate between the strains. We first verified that the *erm* and *tet* resistance markers did not impact *in vivo* colonization of erythromycin-resistant WT (WT-*erm*) and tetracyclin-resistant WT (WT-*tet*) (Fig. 5A). We then showed that both the *ΔCPS4* and *ΔBT2934-38* mutants were outcompeted by the WT strain in two-strain cocolonization experiments (Fig. 5B and C), even though both *ΔCPS4* and *ΔBT2934-38* formed more biofilm than the WT (Fig. 1C; see also Fig. S5 in the supplemental material). When we tested colonization of the double mutant *ΔCPS4 ΔBT2934-2938* against the *ΔCPS4* mutant, we found that they colonized mice similarly (Fig. 5D), indicating that *BT2934* is necessary for colonization only in the WT but not in the *ΔCPS4* background. Taken together, these results showed that increased *in vitro* biofilm formation capacity is not predictive of *in vivo* colonization capacity.

## DISCUSSION

In contrast to oral *Bacteroidales*, intestinal *Bacteroidales* species possess numerous capsular polysaccharide loci that play important beneficial roles during gut colonization, ranging from protecting bacteria from stresses to mediating interactions with the host immune system (17, 20, 24–26). In this study, we showed that deletion of one of B. thetaiotaomicron’s 8 capsular polysaccharides, CPS4, promotes biofilm formation *in vitro*, indicating that capsules mediate yet another important aspect of bacterial physiology.

Bacterial capsular polysaccharides are known to negatively affect biofilm formation by masking surface structures involved in adhesion in many bacteria (27–31). It was shown, for instance, that Escherichia coli capsular polysaccharides inhibit adhesion and auto-aggregation by masking the short autotransporter adhesin antigen 43 as well as the type III secretion system required for attachment in enteropathogenic E. coli (EPEC) (32, 33). The fact that CPS4 is the most expressed capsule in the tested laboratory conditions and *in vivo* (20) probably explains why it was the only capsule of B. thetaiotaomicron we identified by random transposon mutagenesis screening for increased biofilm formation.

In an adhering Δ*CPS4* strain, 30% of the bacteria are acapsulated, indicating that occurrence of only a subpopulation of acapsulated cells is enough to induce biofilm formation. In Bacteroides fragilis, acapsular cells were previously shown to aggregate (17, 34), and we also observed that a completely acapsular strain of B. thetaiotaomicron lacking all 8 capsules (*ΔCPS1-8*) displays a strong aggregation phenotype. Aggregation and cell-to-cell adhesion is an essential part of the biofilm formation maturation process, and free-floating aggregates and air-liquid interface pellicles formed in the absence of any solid surface are considered bona fide biofilms (35). Although the capacity of acapsular bacteria to bind surfaces was not addressed in this work, we hypothesized that, in the absence of a capsule, cell-to-cell interactions are strong drivers of B. thetaiotaomicron biofilm formation. However, due to the protective roles of *Bacteroides* capsules, acapsular strains are rapidly outcompeted by WT strain in axenic mouse colonization (17, 34, 36). Monitoring the expression of the 8 B. thetaiotaomicron capsules by qRT-PCR does not allow detection of acapsular bacteria, as there is no marker of acapsular cells. However, colonization of axenic mice with a mix composed of an acapsular mutant and 8 strains each expressing a single capsule showed that a small amount of acapsular cells was found to persist in the lumen of the small intestine of two out of five mice, potentially due to a decreased immune system pressure allowing the acapsular cells to survive (20). Whether the presence of an acapsular population of bacteria naturally arises and persists in the mammalian gut remains to be demonstrated, and it is still unclear whether increased adhesion capacity of acapsular bacteria is a relevant mechanism by which B. thetaiotaomicron can modify its biofilm formation capacity and biofilm-associated resistance to stress.

*Bacteroides* sp. capsular loci are regulated by a complex transcriptional network involving stochastic inversion of some capsule promoters (17, 37), transcriptional cross-regulation between capsular regulators UpxY and UpxZ (18, 19), and cross talk between polysaccharide utilization loci and capsules through common sigma factors (37). It is also impacted by a range of environmental parameters such as diet, community composition, and host physiology (20, 38, 39). In particular, expression of capsule 4 in mice has been shown to be increased *in vivo* compared to *in vitro* in a high-fiber diet, but it is decreased in the suckling period compared to the weaned period (38, 39), and it is strongly impacted by the immune system (20). Moreover, a transcriptional analysis comparing planktonic cells with biofilms grown on chemostats for 8 days previously showed that CPS4 is downregulated in B. thetaiotaomicron biofilms (15).

Random transposon mutagenesis in the *ΔCPS4* strain identified capsule regulation as the main parameter governing biofilm formation in our conditions. We show that *BT1357*, encoding the UpxZ homolog of *CPS4*, represses transcription of CPS2. UpxZ proteins repress the transcription of nonadjacent capsular operon by interacting with the antiterminator UpxY proteins, necessary for the full transcription of their cognate capsules (18, 19). *BT1357* therefore most likely only interferes with *BT0462*, the UpxY homolog of *CPS2*. Whereas the complex interplay between UpxY and UpxZ homologs of B. fragilis was very well described, it is, to our knowledge, the first description of the precise inhibition pattern of a B. thetaiotaomicron UpxZ homolog (18, 19).

In addition to *BT1357*, we have identified that deletion of *BT2934* impacted capsule production. *BT2934-2947* is the protein O-glycosylation locus of B. thetaiotaomicron (21, 22). This locus is composed of a *wzx* oligosaccharide flippase (*BT2934*) and glycosyl transferases, and its homolog in B. fragilis, the *BF4298-4306* locus, was shown to be required for both *in vivo* and *in vitro* fitness in B. fragilis (21, 22). Accordingly, *BT2934* was previously shown to be important in both *in vitro* and *in vivo* competition experiments between complex communities of B. thetaiotaomicron transposon mutants (23) and was recently described as a putative essential gene (40). Our results confirm both the role of the *ΔBT2934-2938* locus in protein glycosylation and the decreased colonization capacity of a *ΔBT2934-2938* mutant in axenic mice in competition with the WT strain. However, deletion of *BT2934-2938* in the *ΔCPS4* background had no effect on the colonization capacity of this strain. Although we never succeeded in deleting *BT2934* alone, deletion of *BT2934-2938* in the WT and the *ΔCPS4* background did not lead to any growth defect *in vitro*, suggesting that deleting *BT2935-2938* might somehow alleviate the fitness cost associated with the loss of *BT2934*.

We showed that deletion of *BT2934* impacted capsule production independently of protein glycosylation, as complementation by *BT2934* is sufficient to restore the ΔCPS4 biofilm formation phenotype but not the lack of protein glycosylation. The mechanism by which *BT2934* impacts capsule production remains to be elucidated. Because overexpression of *BT2934* in each strain only expressing a single CPS type did not lead to general acapsulation, we hypothesize that *BT2934* does not directly inhibit capsule production. *BT2934* catalyzes the flipping of an oligosaccharide bound to an undecaprenyl-phosphate molecule across the membrane. As oligosaccharide flipping is also required for lipopolysaccharide and capsular synthesis, we speculate that these three processes might compete for undecaprenyl-phosphate or sugar moiety availability. Thus, limiting protein glycosylation by removing *BT2934* could favor the production of some capsules.

While our random transposon mutagenesis in *ΔCPS4* was not saturating, it is surprising that all identified biofilm-deficient mutants corresponded to insertions affecting capsule production rather than a putative adhesion factor unmasked in acapsulated bacteria. This could be indicative of the role played by purely electrostatic interactions between acapsulated bacteria or mediated by multiple and potentially redundant adhesive surface structures.

We show that expression of all capsular polysaccharide of B. thetaiotaomicron hindered biofilm formation, except for CPS8 that, rather, promoted biofilm formation. Consistently, CPS8 expression was shown to be upregulated in 8-day chemostat-grown biofilms (15), while capsules 1, 3, 4, and 6 were downregulated. CPS8 might be either an adhesive capsule or a loose capsule that does not mask adhesion factors. However, if CPS8 did not mask adhesion factors, we would expect the CPS8-only strain to adhere like *ΔCPS1-8*, but CPS8-only formed less biofilm than *ΔCPS1-8*, and it did not aggregate overnight. This suggests that capsule 8 could be a capsule providing adhesion capacity on its own. Interestingly, CPS8 is the only capsular locus of B. thetaiotaomicron containing homologs of FimA, the major component of type V pilus (41). Type V pili are widely found in *Bacteroidetes*, and they were shown to mediate adhesion in Porphyromonas gingivalis (42, 43). Moreover, we previously showed that another homolog of FimA, BT3147, mediated biofilm formation in B. thetaiotaomicron upon truncation of the last 9 amino acids (13). CPS8 is expressed to low levels in axenic mice mono-colonized with B. thetaiotaomicron, and to slightly higher levels in mice colonized with complex communities, suggesting it might confer an advantage to B. thetaiotaomicron when competing with other bacteria for colonization. Whereas a strain expressing only CPS8 is rapidly outcompeted by the WT in the *in vivo* competition experiment, some population of CPS8-only bacteria can be found in the lumen of the small intestine in some mice, reminiscent of the acapsular strain localization (20).

Although several studies described biofilm-like structures in the intestine (44, 45), the relevance of a biofilm organization of the gut microbiota is still controversial. It was nevertheless suggested that surface adhesion, the initial step leading to biofilm formation, could provide benefits for host colonization. Compared to free-floating bacteria, bacteria adhering to food particles could contribute to digestion by optimizing the degradation of complex sugars (46, 47). Moreover, many bacteria were shown to be able to adhere to mucins, the main component of the host mucus layer (48), and this adhesion is believed to be critical to prevent fast shedding of the bacteria with lumenal content (49). Finally, both aggregate and biofilm formation were shown to increase bacterial resistance to several stresses and thus might contribute to bacterial survival in the harsh gut environment (35). In this study, we found no evidence that higher *in vitro* adhesion would lead to better colonization of axenic mice. However, we assessed the abundance of each strain by enumerating bacteria in the feces of mice, even though feces composition only partially recapitulates gut microbiota composition (50). In particular, we can imagine that cells with higher adhesion would not be shed in the feces as much as cells with low adhesion, mimicking a colonization defect. Moreover, besides biofilm formation, *CPS4* and *BT2934* participate in other significant processes, i.e., interactions with the immune system and protein glycosylation, respectively. Therefore, we cannot exclude that the loss of *in vivo* fitness of *ΔCPS4* and *ΔBT2934-38* strains might be due to the the consequence of these deletions on non-biofilm-related functions, overriding any benefit coming from increased biofilm formation.

In this study, we have shown that capsule regulation is a major determinant of biofilm formation. We described a transcriptional inhibition of *CPS2* by the UpxZ homolog of the *CPS4* locus and showed that competition between protein glycosylation and capsule production could constitute another layer of an already very complex capsule regulatory system. Further investigation of the mechanisms of biofilm formation in the gut commensal B. thetaiotaomicron will allow us to address the physiological adaptations of these bacteria within an anaerobic biofilm.

## MATERIALS AND METHODS

### Bacterial strains and growth conditions.

The bacterial strains used in this study are listed in Table S2. B. thetaiotaomicron was grown in brain heart infusion salt (BHIS) broth (51) supplemented with erythromycin (15 μg/ml [erm]), tetracycline (2.5 μg/ml [tet]), gentamicin (200 μg/ml [genta]), or 5′-fluoro-2’-deoxyruidin (200 μg/ml [FdUR]) when required and incubated at 37°C in anaerobic conditions using jars with anaerobic atmosphere generators (GENbag anaerobic; bioMérieux, ref. 45534) or in a C400M Ruskinn anaerobic-microaerophilic station. Escherichia coli S17λpir was grown in Miller’s lysogeny broth (LB) (Corning) supplemented with ampicillin (100 μg/ml) when required and incubated at 37°C with 180 rpm shaking. Cultures on solid medium were done in BHIS broth with 1.5% agar, and antibiotics were added when needed. Bacteria were always streaked from glycerol stock on BHIS agar before being grown in liquid cultures. All media and chemicals were purchased from Sigma-Aldrich unless indicated otherwise. All experiments and genetic constructions of B. thetaiotaomicron were made in the VPI-5482*Δtdk* strain, which was developed for a 2-step selection procedure of unmarked gene deletion by allelic exchange as previously described (52). Therefore, the VPI-5482*Δtdk* is referred to as the wild type in this study.

10.1128/mBio.00729-20.7 TABLE S2 List of strains and plasmids used in this study. Download Table S2, PDF file, 0.2 MB. Copyright © 2020 Bechon et al. 2020 Bechon et al. This content is distributed under the terms of the Creative Commons Attribution 4.0 International license.

### 96-well crystal violet biofilm formation assay.

Overnight culture was diluted to an optical density at 600 nm (OD_600_) of 0.05 in 100 μl BHIS broth and inoculated in technical duplicates in polystyrene Greiner round-bottom 96-well plates. The wells at the border of the plates were filled with 200 μl water to prevent evaporation. Incubation was done at 37°C in anaerobic conditions for 48 h. Then, 25 μl of Bouin solution (picric acid 0.9%, formaldehyde 9%, and acetic acid 5%; HT10132, Sigma-Aldrich) was added directly to each well before removal of the supernatant to prevent the washing out of large aggregates. Cultures were fixed for 10 min, and then the wells were washed once with water by immersion and flicking, and the biofilm was stained with 125 μl 1% crystal violet (V5265; Sigma-Aldrich) for 10 min. Crystal violet solution was removed by flicking, and biofilms were washed twice with water. Stained biofilms were resuspended in a 1:4 acetone:ethanol mix, and absorbance at 575 nm was measured using a Tecan infinite M200 PRO plate reader.

### Targeted mutagenesis.

Deletion mutants were constructed using the previously described vector for allelic exchange in B. thetaiotaomicron—pExchange*-tdk* (52). A list of all the primers used in this study can be found in Table S3. Briefly, a 1-kb region upstream and downstream of the target sequence and pExchange-*tdk* were amplified by PCR using Phusion Flash high-fidelity PCR master mix (Thermo Fischer Scientific; F548). All three fragments were ligated using a Gibson assembly as follows: the inserts and the plasmids were mixed with Gibson master mix 2× (100 μl 5× ISO buffer, 0.2 μl 10,000 U/ml T5 exonuclease [NEB catalog number M0363S], 6.25 μl 2,000 U/ml Phusion HF polymerase [NEB catalog number M0530S], 50 μl 40,000 U/ml *Taq* DNA ligase [NEB catalog number M0208S], and 87 μl dH2O for 24 reactions) and incubated at 50°C for 35 min. The resulting mix was transformed in E. coli S17λpir that was used to deliver the vector to B. thetaiotaomicron by conjugation. Conjugation was carried out by mixing exponentially grown cultures (OD_600_ = 0.6) of the donor and the recipient strain in a 2:1 ratio. The mixture was spotted on BHIS-agar plates and incubated at 37°C in aerobic conditions overnight. The mix was then streaked on BHIS agar supplemented with antibiotic—for selection of B. thetaiotaomicron transconjugants that had undergone the first recombination event—and gentamicin to ensure exclusion of any E. coli growth. Eight of the resulting colonies were grown overnight in BHIS broth with no antibiotic to allow a second recombination event, and the culture was plated on BHIS agar plates supplemented with FdUR to select for loss of plasmid. The resulting deletion mutants were confirmed by PCR and sequencing.

10.1128/mBio.00729-20.8 TABLE S3 Primers used in this study. Download Table S3, PDF file, 0.1 MB. Copyright © 2020 Bechon et al. 2020 Bechon et al. This content is distributed under the terms of the Creative Commons Attribution 4.0 International license.

We used the pNBU2-bla-erm vector (53) for complementation, which inserts in the 5′ untranslated region of the tRNA-Ser, in which we previously cloned the constitutive promoter of *BT1311* encoding the sigma factor RpoD (13). We constructed a pNBU2-bla-tet vector by replacing the erythromycin resistance gene by a tetracycline resistance gene from the pExchange-tet plasmid using Gibson assembly (see above). Target genes were amplified by PCR using Phusion Flash high-fidelity PCR master mix from start codon to stop codon, and they were cloned after *BT1311* promoter by Gibson assembly. The Gibson mix was transformed in E. coli S17λpir, and the resulting E. coli was used to transfer the plasmid to B. thetaiotaomicron by conjugation (see above).

### Transposon mutagenesis.

pSAMbt, the previously published tool for random mariner-based transposon mutagenesis in B. thetaiotaomicron (23), was conjugated in B. thetaiotaomicron as described above. After streaking on BHIS-erm-genta agar plates, isolated colonies were resuspended in 100 μl BHIS broth in 96-well plates, grown overnight, and tested for biofilm formation as described above. The selected clones were then streaked on a fresh BHIS-erm-genta agar plate, and 3 isolated colonies were tested for biofilm formation to ensure that no mix of transposon mutants had occurred during preparation of the library. The genomic DNA of the validated clones was extracted using a DNeasy blood and tissue kit (Qiagen) and sent for whole-genome sequencing at the Mutualized Platform for Microbiology of the Institut Pasteur.

### Electronic microscopy and numbering of acapsulated bacteria.

Overnight cultures were adjusted to 1 ml and an OD_600_ of 1.5. Cells were treated as described by Jacques and Foiry (54) for capsule observation as follows: cultures were resuspended in 5% glutaraldehyde in 0.1 M cacodylate buffer (pH 7.2) and incubated at room temperature for 2 h. Cells were then washed three times in 0.1 M cacodylate buffer (pH 7.2) and fixed 30 min in 1 mg/ml ferritin in 0.1 M cacodylate buffer (pH 7.2). Cells were washed one last time in 0.1 M cacodylate buffer (pH 7.2) and sent for transmission electronic microscopy at the electronic microscopy platform IBiSA of the University of Tours (https://microscopies.med.univ-tours.fr/). Acapsulated cells were counted by hand using the Fiji cell counter plugin.

### India ink stain.

For the India ink stain, 5 μl of overnight cultures were mixed with 3 μl India ink directly on a Superfrost Plus glass microscopy slide (Thermo Fischer Scientific) and left to dry for 2 min. The excess liquid was removed with a paper towel after addition of the coverslip, and the cells were observed with a photonic microscope at ×1000 magnification.

### Observation of aggregates by light microscopy.

First, 1 μl of the bottom of an overnight sedimented culture were mixed with 3 μl phosphate buffered saline (PBS) buffer and dropped on a on Superfrost Plus glass microscopy slide (Thermo Fischer Scientific), and then it was covered with a coverslip. The samples were observed with a photonic microscope at ×400 magnification.

### RNA extraction.

Overnight cultures were mixed with RNAprotect bacteria reagent (Qiagen) at a 1:2 volume ratio. The mix was incubated for 5 min at room temperature and then spun down for 10 min at 5,000 × *g*. The pellet was kept at –80°C. RNA was extracted from the pellet using a FastRNA Pro Blue kit (MP). The pellet was resuspended in 1 ml RNApro and mixed with lysing matrix B. Cells were broken using FastPrep instrument at 40 s, speed 6, twice at 4°C. The lysate was centrifuged for 10 min at 4°C at 12,000 × *g*, and the supernatant was collected and mixed with 300 μl of chloroform. After 5 min of incubation at room temperature, the mix was centrifuged at 12,000 × *g* at 4°C for 5 min. The upper phase was transferred to a tube containing 500 μl cold 100% ethanol, and the nucleic acids were precipitated for 1 h at –20°C. The tubes were centrifuged at 12,000 × *g* at 4°C for 15 min, and the pellet was washed in 500 μl cold 75% ethanol. After centrifugation at 12,000 × *g* and 4°C for 5 min, the ethanol was removed, and the pellet was air-dried. Then, 60 μl of RNA-free water was added to resuspend the nucleic acid, and we treated it with Turbo DNase from the Turbo DNA-free kit (Thermo Fischer Scientific; product number AM1907) for 1.5 h. Then, the enzyme was inactivated using Turbo DNase inactivator for 2 min at room temperature, and the extracted RNA was kept at –20°C.

### qRT-PCR.

We performed reverse transcription using the first-strand cDNA synthesis kit for RT-PCR (AMV) (Sigma-Aldrich) and the protocol described by the supplier. Briefly, 500 μg RNA previously boiled for 15 min at 65°C was mixed with 2 μl 10× reaction buffer, 4 μl MgCl_2_ 25 mM, 2 μl dNTP mix at 10 mM each, 1 μl 3′ primer 20 μM, 1 μl RNase inhibitor 50 U/μl, and 0.8 AMV reverse transcriptase and water. The mix was incubated at 42°C for 1.5 h, and the enzyme was inactivated by heating to 99°C for 5 min. qPCR was performed using SYBR green PCR master mix (Life Technologies). cDNA was mixed with SYBR green master mix as described by the supplier and with corresponding primers in technical duplicates in 384-well plates. The qPCR was performed using the QuantStudio 6 Flex real-time PCR system (Thermo Fischer Scientific) and the “ΔΔCt method” program. We followed 16s rRNA and RpoB as housekeeping genes for normalization.

### Staining of glycosylated proteins.

Overnight cultures were adjusted to 1 ml at an OD_600_ of 1, spun down and resuspended in 100 μl 1× Laemmli-β-mercaptoethanol lysis buffer (Bio-Rad), and boiled for 5 min at 95°C. Then, 10 μl was run on Mini-PROTEAN TGX stain-free TM precast gels (Bio-Rad) in 1× TGX buffer for 40 min at 170 V. The gel was then stained using a Pro-Q Emerald 300 staining for glycoproteins kit (Invitrogen) following the procedure described by the supplier.

### Cocolonization of axenic mice.

Animal experiments were done at the Animalerie Axénique de MICALIS (ANAXEM) platform (Microbiologie de l’Alimentation au Service de la Santé [MICALIS], Jouy-en-Josas, France) according to official authorization number 3441-2016010614307552 delivered by the French Ministry of Education Nationale, Enseignement Supérieur et Recherche. The protocol was approved by a local ethics committee on animal experimentation (committee number 45). All animals were housed in flexible-film isolators (Getinge-La Calhène, Vendôme, France) with controlled environmental conditions (light/dark cycle of 12 h/12 h, temperature between 20 and 22°C, humidity between 45% and 55%). Mice were provided with sterile tap water and a gamma-irradiated standard diet (R03-40; S.A.F.E., Augy, France) *ad libitum*. Their bedding was composed of wood shavings, and they were also given cellulose sheets as enrichment.

For each combination performed in separate isolators, groups of 5 male C3H/HeN germfree mice (6 to 11 weeks old) were gavaged with 200 μl bacterial suspensions containing 100 cells of each of the two strains we coinoculated. One of the strains was conjugated with the pNBU2-bla-erm plasmid, and the other, with the pNBU2-bla-tet plasmid so that they contained a different antibiotic resistance marker for later distinction. One of the combinations (WT [ermR] versus ΔCPS4 [tetR]) was performed twice (10 mice total). We plated the mix used for gavage onto BHIS agar plates with erythromycin or with tetracycline to check the initial ratio. When it was not 1:1, we corrected the measured abundance according to the ratio we had effectively used. At 24, 48, and 72 h after inoculation, feces were collected, split into two tubes, and weighed. One of the tubes was homogenized in 1 ml of BHIS, and serial dilutions were plated onto BHIS agar plates with erythromycin or with tetracycline. The abundance of each strain in the feces was measured by counting the CFU growing on each type of plate using an automated plater (easySpiral; Interscience, France) and counter (Scan500; Interscience, France). The other tube was dried in a speed vac concentrator (Savant, USA) and weighed. This allowed us to calculate the percentage humidity of the feces we were using for each mouse and each condition and to infer the dry weight of the feces used for CFU numbering. We divided the number of bacteria obtained by CFU counting by the dry weight of the feces they were collected from.

### Growth curve.

Overnight cultures were diluted to 0.05 OD_600_ in 200 μl BHIS, which had previously been incubated in anaerobic conditions overnight to remove dissolved oxygen, in Greiner flat-bottom 96-well plates. A plastic adhesive film (adhesive sealing sheet; Thermo Fisher Scientific, AB0558) was added on top of the plate inside the anaerobic station, and the plates were then incubated in a Tecan Infinite M200 Pro spectrophotometer for 24 h at 37°C. The OD_600_ was measured every 30 min after 900 s orbital shaking of 2 mm amplitude.

### Statistical analyses.

Statistical analyses were performed using either R and Rstudio software or GraphPad Prism 8 for Mac OS (GraphPad Software, Inc.). We used only nonparametric tests. For *in vivo* experiments, 5 to 10 mice were used in 1 or 2 independent experiments. For all other experiments, at least 6 biological replicates in at least 2 independent experiments were used. A cutoff *P* value of 5% was used for all tests. *, *P* < 0.05; **, *P* < 0.05; ***, *P* < 0.005.

10.1128/mBio.00729-20.5 FIG S5 *BT2934-2938* impacts biofilm formation. (A) The 96-well plate crystal violet biofilm assay after 48 h of growth in BHIS. The mean of the WT is adjusted to 100%. In the min-max boxplot of 6 to 18 biological replicates for each strain, each replicate is the mean of two technical replicates. *, *P* < 0.05; **, *P* < 0.005; ***, *P* < 0.0005; Mann-Whitney test. The pictures shown under the boxplot in panel C correspond to representative CV-stained microtiter wells after resuspension of the biofilm. Download FIG S5, TIF file, 2.7 MB. Copyright © 2020 Bechon et al. 2020 Bechon et al. This content is distributed under the terms of the Creative Commons Attribution 4.0 International license.
