# Supplementary material for: Capsular Polysaccharide Cross-Regulation Modulates Bacteroides thetaiotaomicron Biofilm Formation
Source: mBio. 2020 Jun 23;11(3):e00729-20. doi: 10.1128/mBio.00729-20 (PMC7315117; doi:10.1128/mBio.00729-20)
Supplement: TABLE S1 [file mBio.00729-20-st001.pdf]

Supplementary Table S1. **Transposon insertion targets.**

**Name**	**Insertion**	**Gene annotation on NCBI**	**Function**	**Reference**
Transposon mutant in WT				
1C1	*BT1356*	capsule polysaccharide export protein	CPS4 synthesis	Xu *et al*., 2003
2C7	*BT1342*	UDP-glucuronic acid epimerase	CPS4 synthesis	Xu *et al*., 2003
3B12	*BT1341*	UDP-glucose 6-dehydrogenase	CPS4 synthesis	Xu *et al*., 2003
1G11	*BT1353*	glycosyltransferase	CPS4 synthesis	Xu *et al*., 2003
1F11	*BT1352*	glycosyltransferase	CPS4 synthesis	Xu *et al*., 2003
Transposon mutant in *ΔCPS4*				
1G8	*BT1358*	transcriptional regulator UpxY homolog	capsule regulation	Chatzidaki-Livanis *et al*., 2010
23A12	*BT1358* // *BT1357* intergenic region	transcriptional regulator UpxY homolog // UpxZ homolog	capsule regulation	Chatzidaki-Livanis *et al*., 2010
42B5	*BT1358*	transcriptional regulator UpxY homolog	capsule regulation	Chatzidaki-Livanis *et al*., 2010
38G6	*BT1358*	transcriptional regulator UpxY homolog	capsule regulation	Chatzidaki-Livanis *et al*., 2010
41C9	*BT1358*	transcriptional regulator UpxY homolog	capsule regulation	Chatzidaki-Livanis *et al*., 2010
34H5	*BT2934*	wzx homolog, LPS biosynthesis transmembrane transport protein, Multidrug and toxin extrusion (MATE) domain	protein O-glycosylation	Fletcher *et al*., 2009

1. Xu J*, et al.* (2003) A genomic view of the human-Bacteroides thetaiotaomicron symbiosis.

2. Chatzidaki-Livanis M, Weinacht KG, & Comstock LE (2010) Trans locus inhibitors limit concomitant polysaccharide synthesis in the human gut symbiont Bacteroides fragilis. *Proceedings of the National Academy of Sciences of the United States of America* 107(26):11976-11980.

3. Fletcher CM, Coyne MJ, Villa OF, Chatzidaki-Livanis M, & Comstock LE (2009) A general O-glycosylation system important to the physiology of a major human intestinal symbiont. *Cell* 137(2):321-331.

4. Martens EC, Roth R, Heuser JE, & Gordon JI (2009) Coordinate regulation of glycan degradation and polysaccharide capsule biosynthesis by a prominent human gut symbiont. *The Journal of biological chemistry* 284(27):18445-18457.
