# Supplementary figures and images for: Capsular Polysaccharide Cross-Regulation Modulates Bacteroides thetaiotaomicron Biofilm Formation
Source: mBio. 2020 Jun 23;11(3):e00729-20. doi: 10.1128/mBio.00729-20 (PMC7315117; doi:10.1128/mBio.00729-20)

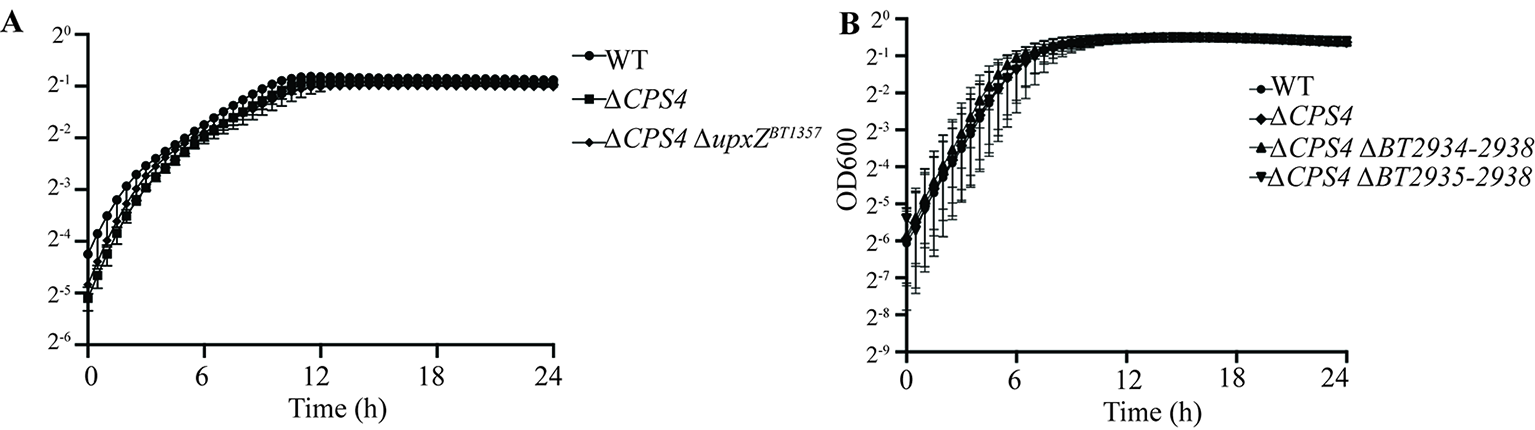

Supplement: FIG S1 [file mBio.00729-20-sf001.tif]

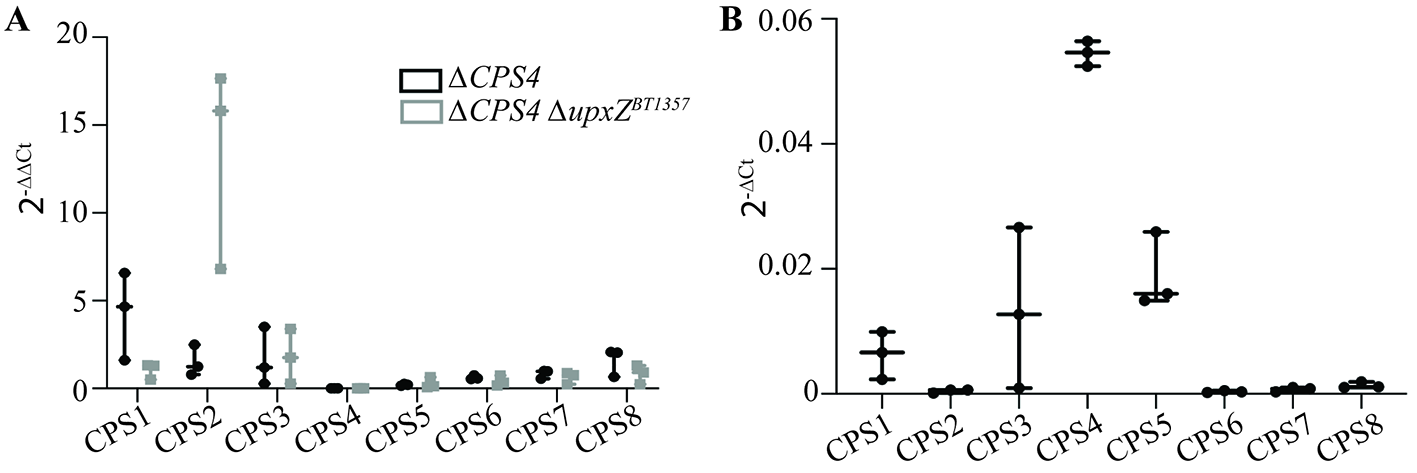

Supplement: FIG S2 [file mBio.00729-20-sf002.tif]

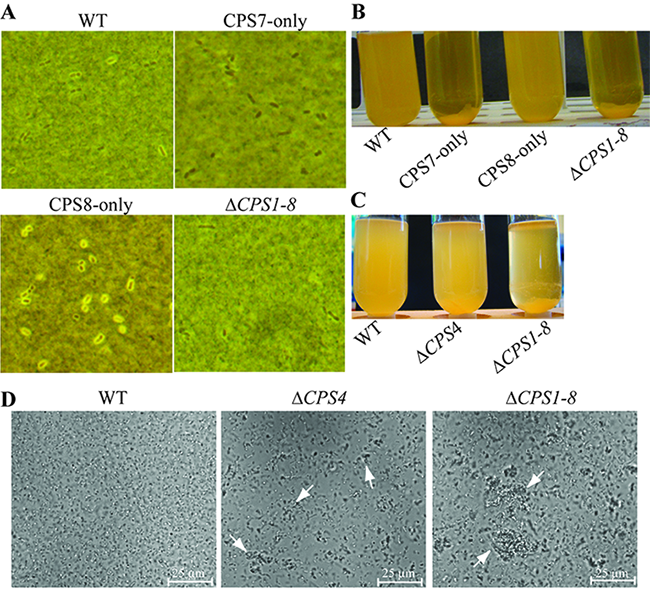

Supplement: FIG S3 [file mBio.00729-20-sf003.tif]

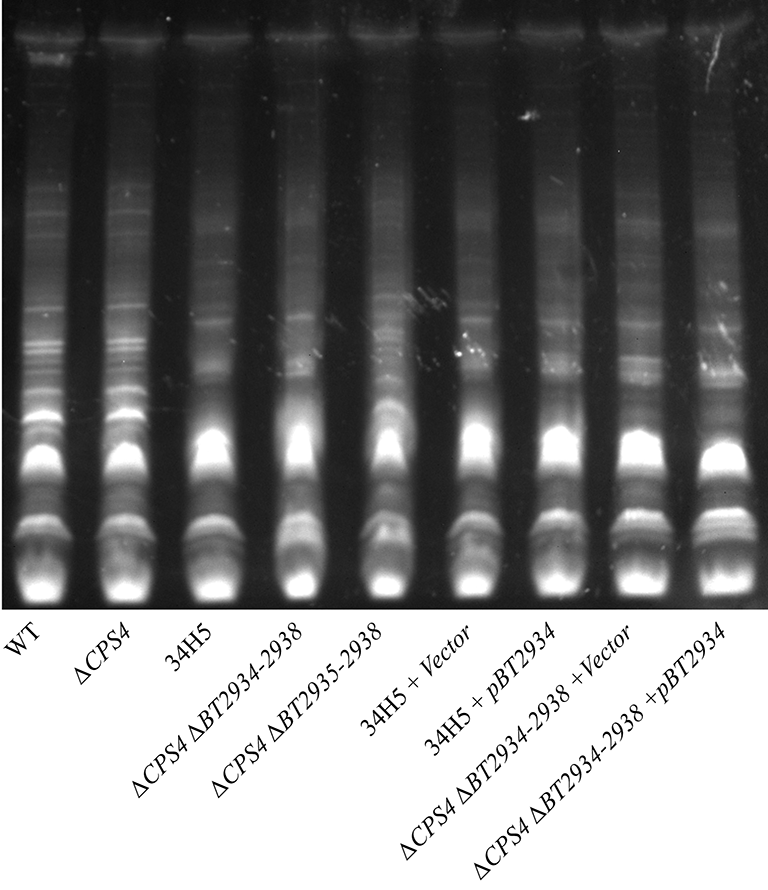

Supplement: FIG S4 [file mBio.00729-20-sf004.tif]

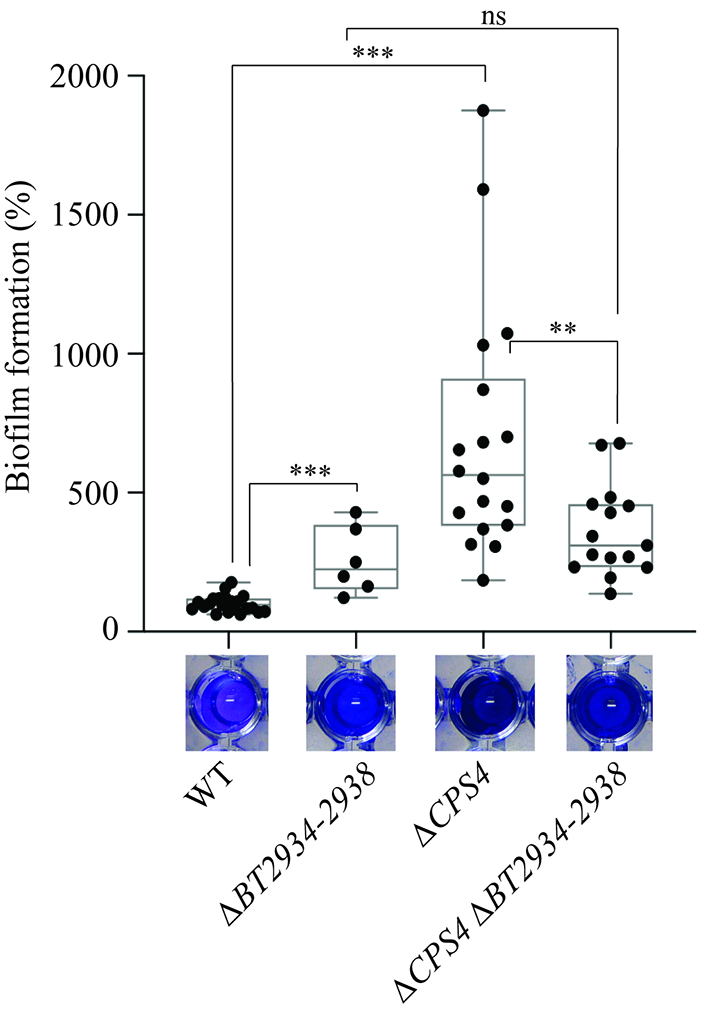

Supplement: FIG S5 [file mBio.00729-20-sf005.tif]
