# Supplementary material for: Capsular Polysaccharide Cross-Regulation Modulates Bacteroides thetaiotaomicron Biofilm Formation
Source: mBio. 2020 Jun 23;11(3):e00729-20. doi: 10.1128/mBio.00729-20 (PMC7315117; doi:10.1128/mBio.00729-20)
Supplement: TABLE S2 [file mBio.00729-20-st002.pdf]

Supplementary Table S2. **List of strains and plasmids used in this study.**

**Name in this paper**	**Genotype**	**Reference**
Bacteroides thetaiotaomicron		
WT	VPI 5482 _*Δtdk*	Koropatkin *et al.*, 2008
1C1	VPI 5482 _*Δtdk*- BT1356::Tn	This study
2C7	VPI 5482 _*Δtdk*- BT1342::Tn	This study
3B12	VPI 5482 _*Δtdk*- BT1341::Tn	This study
1G11	VPI 5482 _*Δtdk*- BT1353::Tn	This study
1F11	VPI 5482 _*Δtdk*- BT1352::Tn	This study
Δ*CPS4*	VPI 5482 _*Δtdk*_ΔBT1356-38	This study
CPS1-only	VPI 5482 _*Δtdk*_*Δcps2-8 cps1-lock*	Porter et al. 2017
CPS2-only	VPI 5482 _*Δtdk*_*Δcps1*_*Δcps3-8*	Porter et al. 2017
CPS3-only	VPI 5482 _*Δtdk*_*Δcps1-2*_*Δcps4-8 cps3-lock*	Porter et al. 2017
CPS4-only	VPI 5482 _*Δtdk*_*Δcps1-3*_*Δcps5-8*	Porter et al. 2017
CPS5-only	VPI 5482 _*Δtdk*_*Δcps1-4*_*Δcps6-8 cps5-lock*	Porter et al. 2017
CPS6-only	VPI 5482 _*Δtdk*_*Δcps1-5*_*Δcps7-8 cps6-lock*	Porter et al. 2017
CPS7-only	VPI 5482 _*Δtdk*_*Δcps1-6*_*Δcps8*	Porter et al. 2017
CPS8-only	VPI 5482 _*Δtdk*_*Δcps1-7 cps8-lock*	Porter et al. 2017
Δ*CPS1-8*	VPI 5482 _*Δtdk*_*Δcps1-8*	Porter et al. 2017
Δ*CPS4*Δ*CPS7*	VPI 5482 _*Δtdk*_*ΔBT1356-38*_*ΔBT2886-62*	This study
Δ*CPS4*Δ*CPS8*	VPI 5482 _*Δtdk*_*ΔBT1356-38*_*ΔBT0038-68*	This study
1G8	VPI 5482 _*Δtdk*_*ΔBT1356-38-BT1358*::Tn	This study
23A12	VPI 5482 _*Δtdk*_*ΔBT1356-38-BT1358-7* intergenic region::Tn	This study
42B5	VPI 5482 _*Δtdk*_*ΔBT1356-38-BT1358*::Tn	This study
38G6	VPI 5482 _*Δtdk*_*ΔBT1356-38-BT1358*::Tn	This study
41C9	VPI 5482 _*Δtdk*_*ΔBT1356-38-BT1358*::Tn	This study
34H5	VPI 5482 _*Δtdk*_*ΔBT1356-38-BT2934*::Tn	This study
Δ*upxZ*^{BT1357}Δ*CPS4*	VPI 5482 _*Δtdk*_*ΔBT1356-38*_*ΔBT1357*	This study
upxY^{BT1358}Δ*CPS4*	VPI 5482 _*Δtdk*_*ΔBT1356-38*_*ΔBT1358*	This study
Δ*upxY*^{BT1358}-Δ*upxZ*^{BT1357}Δ*CPS4*	VPI 5482 _*Δtdk*_*ΔBT1356-38*_*ΔBT1358-7*	This study
Δ*upxZ*^{BT1357}Δ*CPS4*Δ*CPS2*	VPI 5482 _*Δtdk*_*ΔBT1356-38*_*ΔBT1357*_*ΔBT0463-82*	This study
Δ*upxY*^{BT1358}-Δ*upxZ*^{BT1357}Δ*CPS4* + *vector*	VPI 5482 _*Δtdk*_*ΔBT1356-38*_*ΔBT1358-7* + pNBU2-bla-erm-p1311	This study
Δ*upxY*^{BT1358}-Δ*upxZ*^{BT1357}Δ*CPS4* + *pupxZ*^{BT1357}	VPI 5482 _*Δtdk*_*ΔBT1356-38*_*ΔBT1358-7* + pNBU2-bla-erm-p1311-*BT1357*	This study

Δ*upxZ*^{BT1357}Δ*CPS4 + vector*	VPI 5482 *_Δtdk_ΔBT1356-38_ΔBT1357* + pNBU2-bla-erm-p1311	This study
Δ*upxZ*^{BT1357}Δ*CPS4 + pupxZ*^{BT1357}	VPI 5482 *_Δtdk_ΔBT1356-38_ΔBT1357* + pNBU2-bla-erm-p1311-*BT1357*	This study
Δ*CPS4*Δ*BT2934-2938*	VPI 5482 *_Δtdk_ΔBT1356-38_ΔBT2934-8*	This study
Δ*CPS4*Δ*BT2935-2938*	VPI 5482 *_Δtdk_ΔBT1356-38_ΔBT2935-8*	This study
Δ*CPS4*Δ*BT2934-2938 + Vector*	VPI 5482 *_Δtdk_ΔBT1356-38_ΔBT2934-8*+ pNBU2-bla-tet-p1311	This study
Δ*CPS4*Δ*BT2934-2938 + pBT2934*	VPI 5482 *_Δtdk_ΔBT1356-38_ΔBT2934-8*+ pNBU2-bla-tet-p1311-*BT2934*	This study
34H5 + *Vector*	VPI 5482 *_Δtdk_ΔBT1356-38_BT2934*::Tn+ pNBU2-bla-tet-p1311	This study
34H5 + *pBT2934*	VPI 5482 *_Δtdk_ΔBT1356-38_BT2934*::Tn+ pNBU2-bla-tet-p1311-*BT2934*	This study
WT-ery	VPI 5482 *_Δtdk*+ pNBU2-bla-ery-p1311	This study
WT-tet	VPI 5482 *_Δtdk*+ pNBU2-bla-tet-p1311	This study
Δ*CPS4-ery*	VPI 5482 *_Δtdk_ΔBT1356-38*+ pNBU2-bla-ery-p1311	This study
Δ*CPS4-tet*	VPI 5482 *_Δtdk_ΔBT1356-38*+ pNBU2-bla-tet-p1311	This study
Δ*BT2934-2938*	VPI 5482 *_Δtdk_ΔBT2934-8*	This study
Δ*BT2934-2938 tet*	VPI 5482 *_Δtdk_ΔBT2934-8* + pNBU2-bla-tet-p1311	This study
Δ*CPS4*Δ*BT2934-2938 tet*	VPI 5482 *_Δtdk_ΔBT1356-38_ΔBT2934-8*+ pNBU2-bla-tet-p1311	This study
CPS1-only+pBT2934	VPI 5482 *_Δtdk_Δcps2-8 cps1-lock*+ pNBU2-bla-tet-p1311-*BT2934*	This study
CPS2-only+pBT2934	VPI 5482 *_Δtdk_Δcps1_Δcps3-8*+ pNBU2-bla-tet-p1311-*BT2934*	This study
CPS3-only+pBT2934	VPI 5482 *_Δtdk_Δcps1-2_Δcps4-8 cps3-lock*+ pNBU2-bla-tet-p1311-*BT2934*	This study
CPS4-only+pBT2934	VPI 5482 *_Δtdk_Δcps1-3_Δcps5-8*+ pNBU2-bla-tet-p1311-*BT2934*	This study
CPS5-only+pBT2934	VPI 5482 *_Δtdk_Δcps1-4_Δcps6-8 cps5-lock*+ pNBU2-bla-tet-p1311-*BT2934*	This study
CPS6-only+pBT2934	VPI 5482 *_Δtdk_Δcps1-5_Δcps7-8 cps6-lock*+ pNBU2-bla-tet-p1311-*BT2934*	This study
CPS8-only+pBT2934	VPI 5482 *_Δtdk_Δcps1-7 cps8-lock*+ pNBU2-bla-tet-p1311-*BT2934*	This study
Escherichia coli		
S17λpir_pSAM-bt	S17λpir_pSAM-bt	This study
S17λpir_pExchange-tdk	S17λpir_pExchange-tdk	This study
S17λpir_pExchange-BT1356-38	S17λpir_pExchange-BT1356-38	This study
S17λpir_pExchange-BT2886-62	S17λpir_pExchange-BT2886-62	This study
S17λpir_pExchange-BT0038-68	S17λpir_pExchange-BT0038-68	This study
S17λpir_pExchange-BT1357	S17λpir_pExchange-BT1357	This study
S17λpir_pExchange-BT1358	S17λpir_pExchange-BT1358	This study
S17λpir_pExchange-BT1358-7	S17λpir_pExchange-BT1358-7	This study
S17λpir_pExchange-BT0463-82	S17λpir_pExchange-BT0463-82	This study

S17λpir_pExchange-BT2934-8	S17λpir_pExchange-BT2934-8	This study
S17λpir_pExchange-BT2935-8	S17λpir_pExchange-BT2935-8	This study
S17λpir_ pNBU2-bla-erm-p1311	S17λpir_ pNBU2-bla-erm-p1311	This study
S17λpir_pNBU2-bla-erm-p1311-BT1357	S17λpir_pNBU2-bla-erm-p1311-BT1357	This study
S17λpir_pNBU2-bla-tet-p1311	S17λpir_pNBU2-bla-tet-p1311	This study
S17λpir_pNBU2-bla-tet-p1311-BT2934	S17λpir_pNBU2-bla-tet-p1311-BT2934	This study
**Name**	**Description**	**Reference**
**Plasmids**		
pSAM-bt	Random transposon mutagenesis	Goodman *et al.*, 2009
pExchange-tdk	Site-directed unmarked gene deletion	Martens *et al.*, 2008
pExchange-BT1356-38	Site-directed unmarked gene deletion of BT1356-38	This study
pExchange-BT2886-62	Site-directed unmarked gene deletion of BT2882-62	This study
pExchange-BT0038-68	Site-directed unmarked gene deletion of BT0038-68	This study
pExchange-BT1357	Site-directed unmarked gene deletion of BT1357	This study
pExchange-BT1358	Site-directed unmarked gene deletion of BT1358	This study
pExchange-BT1358-7	Site-directed unmarked gene deletion of BT1358-7	This study
pExchange-BT0463-82	Site-directed unmarked gene deletion of BT0463-82	This study
pExchange-BT2934-8	Site-directed unmarked gene deletion of BT2934-8	This study
pExchange-BT2935-8	Site-directed unmarked gene deletion of BT2935-8	This study
pNBU2-bla-erm	Complementation	Wang et al 2000
Vector (erm)	Complementation	This study
pupxZ^{BT1357}	Complementation with BT1357	This study
Vector (tet)	Complementation	This study
pBT2934	Complementation with BT2934	This study

1. Koropatkin NM, Martens EC, Gordon JI, & Smith TJ (2008) Starch catabolism by a prominent human gut symbiont is directed by the recognition of amylose helices. *Structure* 16(7):1105-1115.
2. Porter NT & Martens EC (2017) The Critical Roles of Polysaccharides in Gut Microbial Ecology and Physiology. *Annual review of microbiology* 71:349-369.
3. Goodman AL*, et al.* (2009) Identifying genetic determinants needed to establish a human gut symbiont in its habitat. *Cell host & microbe* 6(3):279-289.
4. Martens EC, Roth R, Heuser JE, & Gordon JI (2009) Coordinate regulation of glycan degradation and polysaccharide capsule biosynthesis by a prominent human gut symbiont. *The Journal of biological chemistry* 284(27):18445-18457.
5. Wang J, Shoemaker NB, Wang G-R, & Salyers AA (2000) Characterization of a Bacteroides Mobilizable Transposon, NBU2, Which Carries a Functional Lincomycin Resistance Gene. *Journal of bacteriology* 182(12):3559-3571.
