# Supplementary material for: Capsular Polysaccharide Cross-Regulation Modulates Bacteroides thetaiotaomicron Biofilm Formation
Source: mBio. 2020 Jun 23;11(3):e00729-20. doi: 10.1128/mBio.00729-20 (PMC7315117; doi:10.1128/mBio.00729-20)
Supplement: TABLE S3 [file mBio.00729-20-st003.pdf]

Supplementary Table S3. **Primers used in this study**

**Construct**	**Name**	**Sequence (5'-->3')**
pExchange	pEx-ch-F	TGGGAATTCCCCTCCACCGC
	pEx-ch-R	GGGGAGAGGACGGACAGAAGAT
	pExchangeR	CGTCGACTCGAATGTTATCTTC
	pExchangeF	TCTAGAGCGGCCGCCACC
pExchange-BT1356-1338	1356-5R	AGAACAAAAAAGTAGAATGCTAAAAAGTCGTTGTATTTTC
	1356-5F	GCGGTCGACAGAATAACGAATAAGTGTGGGG
	1356-3F	GAAAATACAACGACTTTTTAGCATTCTACTTTTTTGTTCTTTCTTAGAATTGAAATAAACG
	1356-3R	CCCTCTAGAATTCGATCCTACGAATACGACC
pExchange-BT0038-0068	cps8-intF	ACGGCATAGATGTAGCCAAAAG
	cps8-intR	GATCGCCCAACCTAGTGTC
	cps8-3F	TTGATAATTCTTTTAAACATGATGCATGGTCTATTACAACCTGTC
	cps8-3R	GCGGTGGCGGCCGCTCTAGACAAAGAAGCTTTCCTTCTGAC
	cps8-5R	GGTTGTAATAGACCATGCATCATGTTTAAAAGAATTATCAATAGAAACATG
	cps8-5F	AGATAACATTCGAGTCGACGCATAAATTAGTTTGAGCGACG
pExchange-BT2886-2862	cps7-extF	GACTGTTCCGAACATTG
	cps7-extR	AGCCTTGCTCAAAAACTGG
	cps7-5R	GTCAGAGAGAAATGATAGCAAATAACTCCTCCAATTCTATCATTTAAAG
	cps7-5F	AGATAACATTCGAGTCGACGGCAGAGTGAACTTTATCCTC
	cps7-3F	ATAGAATTGGAGGAGTTATTTGCTATCATTTCTCTCTGACAGATG
	cps7-3R	GCGGTGGCGGCCGCTCTAGATCCTTAGTCCCTGTACCC
pExchange-BT1358	1358-5F	GCGGTGGCGGCCGCTCTAGACAGCAGCCTCATTCTTTAGTGC
	1358-5R	TTTCACTTATATTTAAACCCCTATTTACCCCACACTTATTCGT
	1358-3F	AATAAGTGTGGGGTAAATAGGGGTTTAAATATAAGTGAAACAAGCA

	1358-3R	AGATAACATTCGAGTCGACGGTTGCATGCACTCATCCG
pExchange-BT1357	1357-3R	AGATAACATTCGAGTCGACGTGATTGTAATCTTGATATGCTAGCAAAG
	1357-3F	ATTAAGTGAATAAAAGGGGTCTTTTTATTTGTCAATGAAAATACAACGAC
	1357-5R	TTCATTGACAAATAAAAAGACCCCTTTTATTCACTTAATGCTTG
	1357-5F	GCGGTGGCGGCCGCTCTAGATGTCAAGTTCAGGTTTCAGGTC
pExchange-BT1358-1357	1357-58 3F	AATAAGTGTGGGGTAAATAGCTTTTTATTTGTCAATGAAAATACAACGAC
	1357-58 5R	TTCATTGACAAATAAAAAGCTATTTACCCCACACTTATTCGT
pExchange-BT0463-0482	cps2-3R	GCGGTGGCGGCCGCTCTAGAGATTGAAAGTGGCGGCAG
	cps2-3F	TGAGTACTAATAACAAATCATCCGGTTCTAAGAATAAACCTGAG
	cps2-5R	GGTTTATTCTTAGAACCGGATGATTTGTTATTAGTACTCAAGATTTGAG
	cps2-5F	AGATAACATTCGAGTCGACGTGAAAGCTGTCAGGAAGCTAAG
pExchange-BT2934-2938	2934-5F	AGATAACATTCGAGTCGACGGGAAAGACTTCCAGGCACG
	2934-5R	GCATTTGGGGACTTCACCGGATTATTATAATCGGTGAAGGAGAGTG
	2938-3F	CCTTCACCGATTATAATAATCCGGTGAAGTCCCCAAATGC
	2938-3R	GCGGTGGCGGCCGCTCTAGATCTCTATGAATGTCCTGTTTTCGT
pExchange-BT2935-2938	2935-5F	AGATAACATTCGAGTCGACGGGATAGCTCCGCGAGCTC
	2935-5R	GCATTTGGGGACTTCACCGGTCATGATATCTTTTTCTTTTTAATGAACTG
	35-2938-3F	AAAAGAAAAAGATATCATGACCGGTGAAGTCCCCAAATGC
pNBU2-bla	pNBU-chR	GCCAATGCACAAATGCTGTTCC
	pNBU-chF	CAGGTGTATTCCCATCCGG
	pNBU-F	CGACGTCGACTAATTGCC
	pNBU-R	ATGTTAAAAACAGATTTGGAGTGC

pNBU2-bla-tetR	pNBU-del-eryF	ATGTCATCAAAATAAAAACAATAGGCCACATGCAAC
	pNBU-del-eryR	ATTTATAATATTCATTATAACCTCTCCTTAATTTATTG
	tetQ-F	ATTAAGGAGAGGTTATAATGAATATTATAAATTTAGGA ATTCTTGCTC
	tetQ-R	TTGCATGTGGCCTATTGTTTTTATTTTGATGACATTGATT TTTGG
	tetQ-NBU-chF	CGGGATGAACCATGAGTAC
	tetQ-NBU-chR	GTACCGAGGACGCGTAAAC
pNBU2-bla-erm-BT1357	pNBU-1357F	TCCAAATCTGTTTTTAACATATGGTGAGTTTTTACTAC AAG
	pNBU-1357R	TAGGCAATTAGTCGACGTCGTTAGTTGGTTTCACACAGT TCC
pNBU2-bla-tet-p1311-BT2934	NBU-2934-F	CACTCCAAATCTGTTTTTAACATATGAGTGAAGAACAGT CACTGAAAC
	NBU-2934-R	GATAGGCAATTAGTCGACGTCGTCATGATATCTTTTTCT TTTTAATGAACTG
qPCR	16SQF	TCAGCTCGTGTTGTGAAATG
	16SQR	GTAAGGGCCATGATGACTTG
	rpoB-Q-F	CAAATTTCACGCCCAAAGTT
	rpoB-Q-R	GTGCGTCAGGAAGATATG
	0381-QF	CGCTTTATCATGTCGTTGGA
	0381-QR	GTACAAGCCGGAGCTTTTTG
	0463-QF	TCCGATCACAAAAGGAGTGA
	0463-QR	ACGTTTAAAGCCCGGAAGAT
	0602-QF	TTGGGTTACATCGGTCTTCC
	0602-QR	TTTGAAGGTGGTGTGACCTG
	1355-QR	CGCAGGTTCTATCACTGCAA
	1355-QF	AGGAGCGATTGCAAAATGAC
	1653-QF	TGTTCCATTGAAAGCTTCAGG
	1653-QR	CTCCCCATCAATATCCAGCTT
	1722-QR	TGCAAAGACTGGCTTTTCCT
	1722-QF	TGAGCAGGAGCGTTTACAGA
	2885-QR	TAGAGGAAGAGTCCCGTTGGT
	2885-QF	GGCAACGCAAATGGATTACTA
	0039-QF	GGCATTTGCTTGTTTTATGGA
	0039-QR	AGGGCTGGTCATATCGTTTCT

3
